# Heat Rate Prediction of Combined Cycle Power Plant Using an Artificial Neural Network (ANN) Method

**DOI:** 10.3390/s21041022

**Published:** 2021-02-03

**Authors:** Yondha Dwika Arferiandi, Wahyu Caesarendra, Herry Nugraha

**Affiliations:** 1 Engineering Department, Cilegon Combined Cycle Power Plant, PT Indonesia Power, Cilegon 42454, Indonesia; 2 Faculty of Integrated Technologies, Universiti Brunei Darussalam, Jalan Tungku Link, Gadong BE1410, Brunei; wahyu.caesarendra@ubd.edu.bn; 3 Research, Innovation and Engineering Department, Head Office, PT Indonesia Power, Jakarta 12950, Indonesia; nugraha.herry@indonesiapower.co.id

**Keywords:** artificial neural network (ANN), combined cycle power plant (CCPP), heat rate prediction

## Abstract

Heat rate of a combined cycle power plant (CCPP) is a parameter that is typically used to assess how efficient a power plant is. In this paper, the CCPP heat rate was predicted using an artificial neural network (ANN) method to support maintenance people in monitoring the efficiency of the CCPP. The ANN method used fuel gas heat input (P1), CO_2_ percentage (P2), and power output (P3) as input parameters. Approximately 4322 actual operation data are generated from the digital control system (DCS) in a year. These data were used for ANN training and prediction. Seven parameter variations were developed to find the best parameter variation to predict heat rate. The model with one input parameter predicted heat rate with regression R^2^ values of 0.925, 0.005, and 0.995 for P1, P2, and P3. Combining two parameters as inputs increased accuracy with regression R^2^ values of 0.970, 0.994, and 0.984 for P1 + P2, P1 + P3, and P2 + P3, respectively. The ANN model that utilized three parameters as input data had the best prediction heat rate data with a regression R^2^ value of 0.995.

## 1. Introduction

Combined cycle power plants (CCPPs) are one of the power plants types that could produce electricity with high efficiency and low air pollutant. However, the CCPPs production cost is relatively higher than the coal-fired power plant due to prime energy costs. Hence, operating CCPPs as efficiently as possible is required to decrease production costs. CCPP highest efficiency could be achieved with maximum load and operating in base load. Unfortunately, energy supply instability is a common problem of CCPPs in Indonesia. This issue leads the CCPP management staff struggling to predict the performance of their CCPP. Assessing historical performance data using machine learning will help to predict the CCPP performance accurately.

Some literature about gas turbine (GT) or CCPP modeling with machine learning has been studied. Wood [1] used the transparent open box (TOB) to predict the combined cycle gas turbine’s power output. Liu et al. [2] predicted gas turbine performance with high dimensional model representation (HDMR) and an artificial neural network (ANN). A distributed control system (DCS) was utilized as a tool to provide selected input data. It was presented that the holistic ANN model had a higher accuracy to predict gas turbine performance. The ANN was utilized to describe the micro gas turbine performance and effectively evaluate it in a real installation in any climate as presented by Bartolini et al. [3]. Anvari et al. [4] utilized an ANN on combined cooling heating and power (CCHP) to predict GT performance prediction. Some underlying parameters were chosen as inputs but did not include any fuel gas characteristics. Fast et al. [5] developed an ANN model to predict the performance of an industrial GT. Relative humidity, ambient pressure and ambient temperature were used as input parameters. It was reported that the best possible modeling of the plant was optimized for 10,000 epochs. Rossi et al. [6] reported ANN modeling for baseline consumption of a combined heat and power plant. Elfaki et al. [7] predicted the electrical output power of CCPP using the regression ANN model. Moreover, ANN has been applied to other GT-based application [8,9,10,11]. ANN is also widely used in power plants with multiple objectives. Smrekar et al. [12] predicted power generated by a coal-fired power plant. Thermal efficiency and power plant pollutant were also predicted with ANNs [13,14,15,16].

To date, there is no actual study predicting CCPP performance using fuel gas characteristics such as gas heat units in million British thermal units (MMBTU) or CO_2_ percentage, even though those two parameters are usually used in the gas purchase agreement between the gas suppliers and power plant operator. Furthermore, the power plant operator usually has a plant efficiency written in their contract with the regulator. Omar et al. [17] showed that decreasing power generation leads to higher specific fuel consumption. 

According to the research gap, this paper proposes a prediction method of CCPP heat rate performance with gas heat unit in million British thermal units (MMBTUs), carbon dioxide (CO_2_) percentage contain in the fuel gas and generated power in megawatts (MW) as input parameters. Some studies above showed that ANN is effective in predicting gas turbine performance. Therefore, an ANN was used as a machine learning tool to predict the power plant heat rate. 

## 2. Materials and Methods

Artificial neural networks are a technology-based on studies of the brain and nervous system. These networks emulate a biological neural network, but they use a reduced set of biological neural systems concepts. Specifically, ANN models simulate the electrical activity of the brain and nervous system. Processing elements (also known as either a neurodes or perceptrons) are connected to other processing elements. Typically, the neurodes are arranged in a layer or vector, with the output of one layer serving as the input to the next layer and possibly other layers. A neurode may be connected to all or a subset of the neurodes in the subsequent layer, with these connections simulating the brain’s synaptic connections. Weighted data signals entering a neurode simulate a nerve cell’s electrical excitation and, consequently, the transference of information within the network or brain. The input values to a processing element are multiplied by a connection weight that simulates the strengthening of neural pathways in the brain. Through the adjustment of the connection strengths or weights, learning is emulated in ANNs [18].

An ANN is comprised of an input layer, hidden layers and an output layer. All input nodes have their weight connection of a neuron. Then the relationship between input parameters and output parameters is learned through the iterative training process. Linear and nonlinear activation functions are included in the ANN system, resulting in a good prediction of nonlinear behavior systems. 

The components of ANNs are neurons, transfer functions and predefined layers. In a multilayer perceptron (MLP), the transfer function used are tan-sigmoid (tansig), log-sigmoid (logsig), and linear purelin functions [19]. Tansig receives the signal in (−∞, +∞) and converts them to output signal within a (−1, +1) range. Logsig receives the input signals in the range of (−∞, +∞) and transfers them to the (0, 1) limit. Pureline is a linear function that maps the input data between (−∞, +∞) and results in the identical data in (−∞, +∞) interval [4]. 

**Figure 1:**
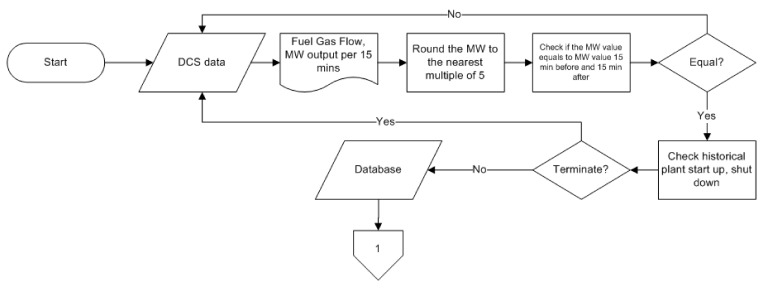

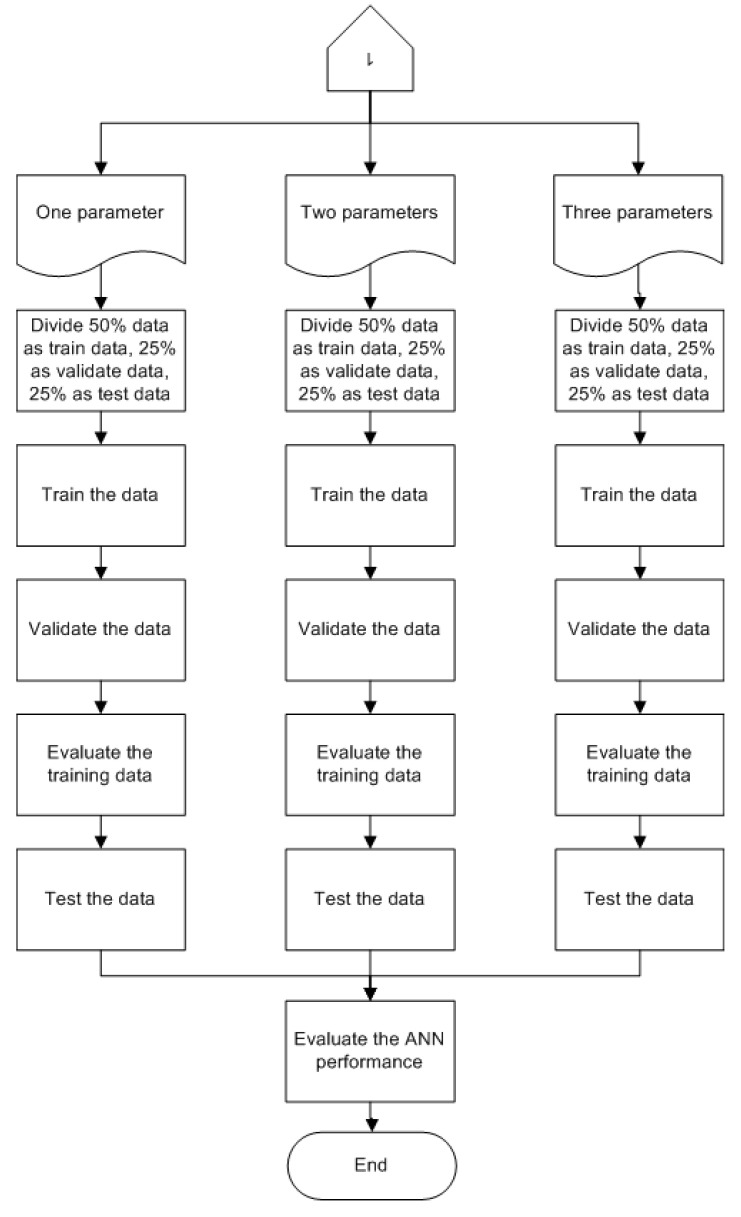
Flowchart of the research method.

Our proposed method for predicting heat rate in a CCPP was developed and demonstrated for a 740 MW CCPP with configuration two GTs, two heat recovery Sseam generators (HRSGs), and one steam turbine. However, this paper only provides predictions with configuration 1-1-1 (one GT and one HRSG stop) due to a lack of gas supply. Real operation data were collected over one year (2019). Figure 1 presents this paper’s flowchart. The input parameters in this paper were MMBTU, CO_2_ percentage and power generated in MW. Total generated power of the CCPP, from a GT and steam turbine, recorded by a distributed control system (DCS) was used as an input parameter in this paper. Since the DCS did not provide a fuel gas heating unit in MMBTUs and CO_2_ percentage, the gas supplier’s hourly data were used. The gas provided by the supplier went to the combustion chamber and was used as a fuel for the GT. The exhaust gas from the process was used to feed the HRSG. Since the total heat from these two processes is equal to the gas supplied, the gas data from supplier was used. Equation (1) was used to calculate the plant heat rate [20].

(1) $$\mathrm{HR}=\frac{\mathrm{Mf}\;x\;\mathrm{HHV}}{P}$$

where: HR = Heat Rate (kcal/kWh); Mf = Fuel gas flow (kNm^3^/h); HHV = High Heating Value (BTU/SCF); P = power output (MW).

One input parameter was used with the ANN to see the result in predicting the heat rate. Two combined parameters were used with the ANN to see the result in predicting the heat rate. And finally, the three combined parameters were used with the ANN to see the result in predicting the heat rate. Fuel gas flow was recorded in a DCS and a High Heating Value (HHV) recorded by the gas suppliers and used in this paper. As shown in Figure 1, the generated power recorded by the DCS was the key to get clean data, as per 15 min of data recorded by the DCS collected and evaluated. The data was normalized by rounding it to the nearest multiple of five, then evaluated by comparing it with data 15 min before and after. If the normalized data had an equal value with data 15 min before and after, the data would continue to the next step. If the value was not equal, then it was terminated. This step was necessary to determine whether the CCPP was in steady-state load condition or not. The measured data were selected carefully to remove the transient during start-up, shut down and load change. Finally, 4322 clean data were provided in steady-state power plant conditions, as presented in Table 1. The input parameters were combined as shown in Table 2 to find the best combination to predict heat rate.

**Figure 2:**
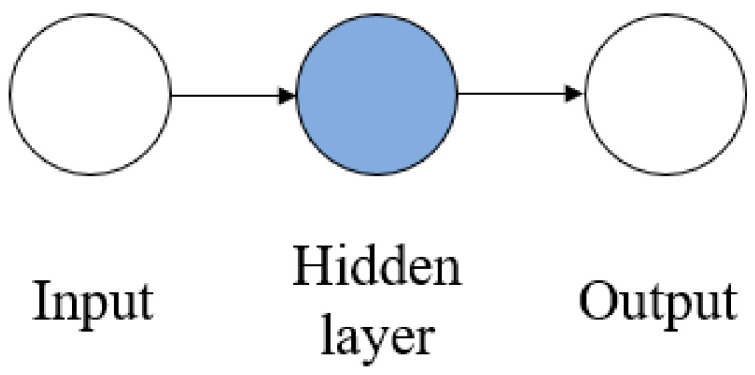
ANN structure for one input parameter (P1, P2 and P3).

A feed-forward backpropagation network type was implemented in all ANN models and trained using gradient descent with momentum and adaptive learning rate backpropagation (traingdx). Mean squared error (MSE) was used as the loss function. With a back-propagation algorithm, the learning function changed the weight between the neurons to reduce the difference. Since success implementation for power plant application has been shown in other studies [2,5,8], the tansig and purelin activation function was implemented in this paper. One hidden layer was used with purelin as a transfer function. The total neuron used was one neuron. ANN structure for the model with one input parameter is shown in Figure 2.

**Figure 3:**
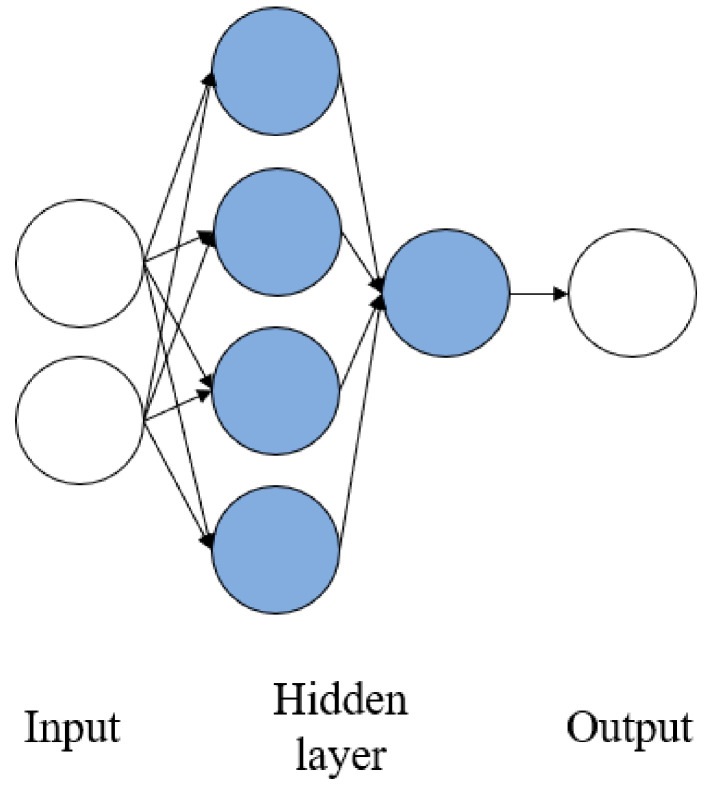
ANN structure for two input parameters (P1 + P2, P1 + P3 and P2 + P3).

Two input parameters were combined as presented in the Table 2. In these models, two hidden layers were used. The first hidden layer used four neurons as a rule of thumb of the hidden layer as the input layer square, and the second hidden layer used 1 neuron. The transfer function used in the first hidden layer was tansig, and the second hidden layer used purelin as a transfer function. The ANN structure for the model with two input parameters is shown in Figure 3.

**Figure 4:**
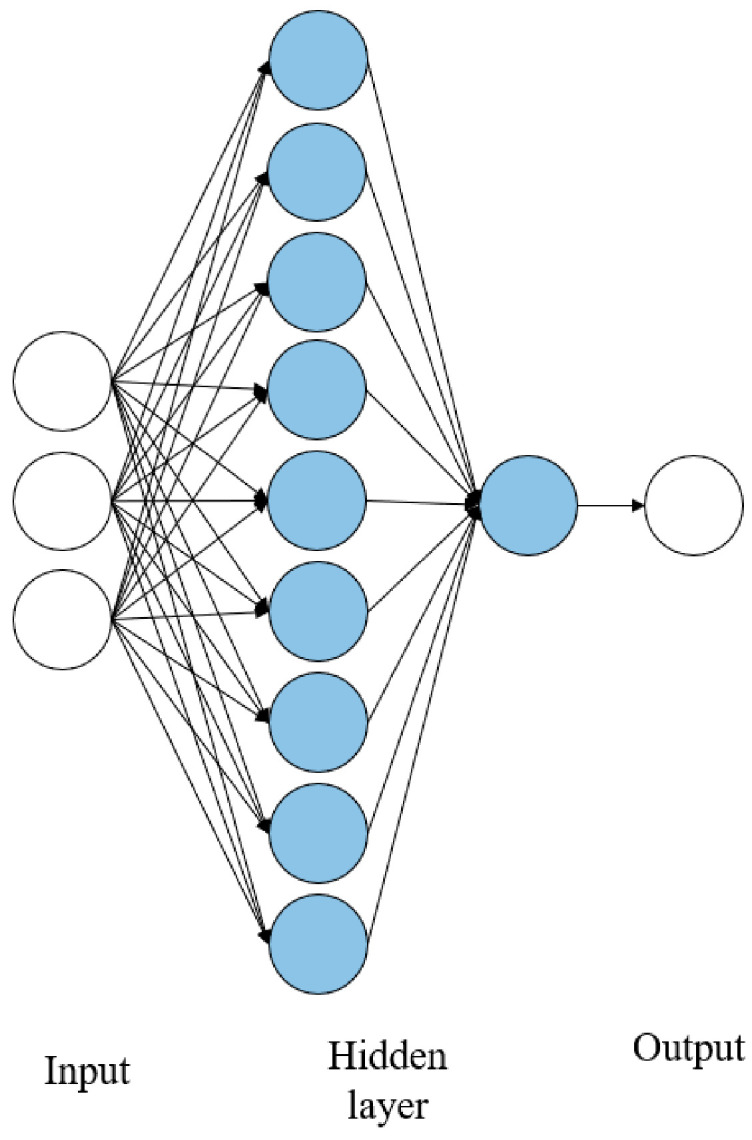
ANN structure for three input parameters (P1 + P2 + P3).

The last model was the combination of all three input parameters. Two hidden layers were used, with the first hidden layer using nine neurons as a rule of thumb of the hidden layer as the input layer square, and the second layer used 1 neuron. The first layer used tansig as a transfer function, and the second layer used purelin as a transfer function. Figure 4 shows the ANN structure for the model with three input parameters:

The output parameter for all models was the plant heat rate, since the plant heat rate was usually used in a contractual agreement to judge whether the power plant was operated efficiently or not. Each model used 10,000 epoch iterations and 1000 validation checks. Data used for training was 50%, while 25% was used for validation and the other 25% used for the test.

## 3. Results

### 3.1. One Input Parameter

**Figure 5:**
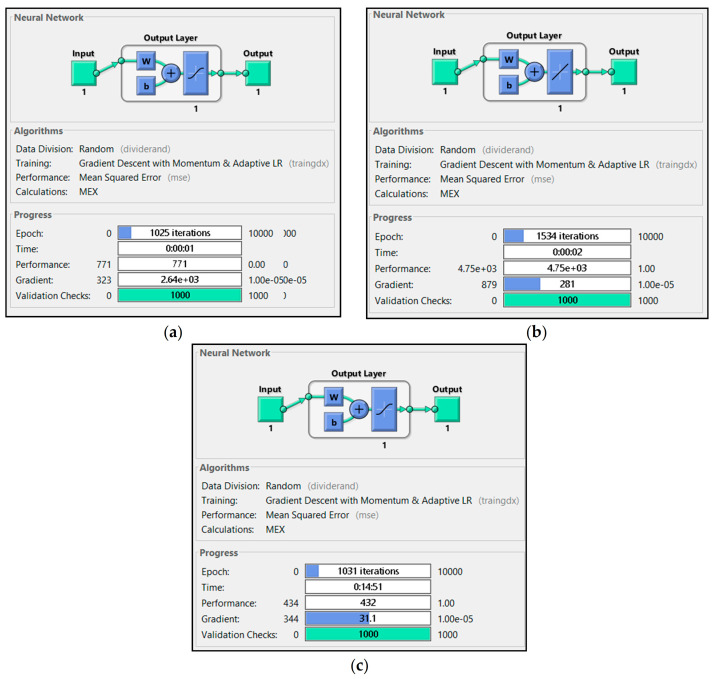
Training results of (**a**) P1, (**b**) P2 and (**c**) P3.

**Figure 6:**
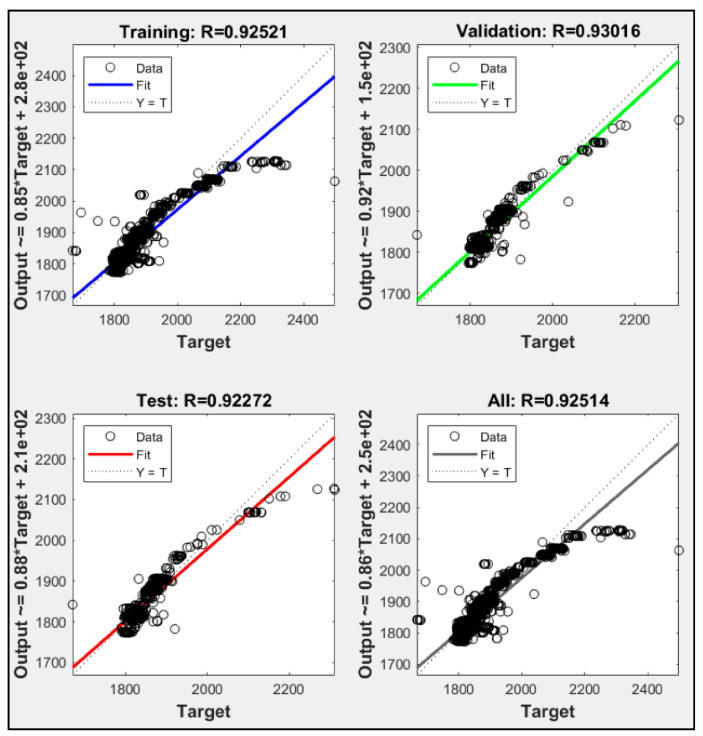
P1 regression result.

**Figure 7:**
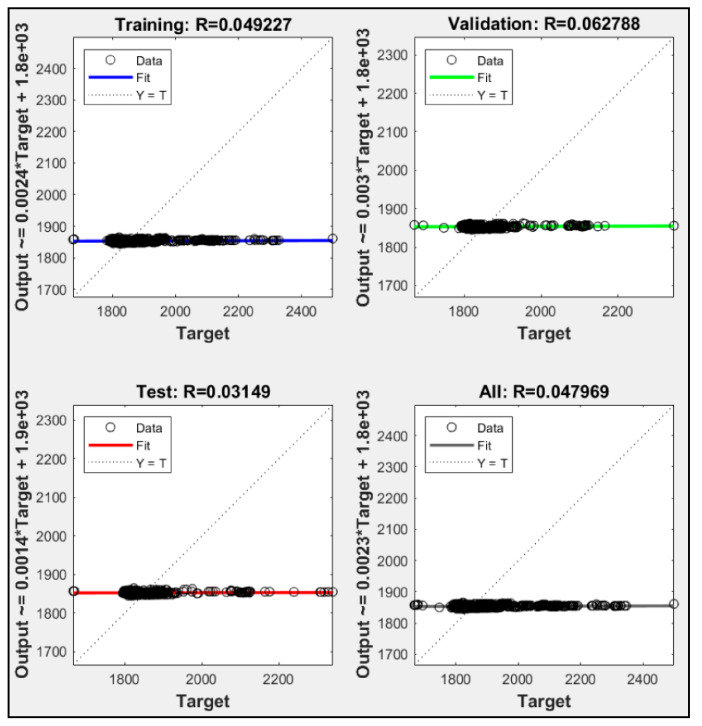
P2 regression result.

**Figure 8:**
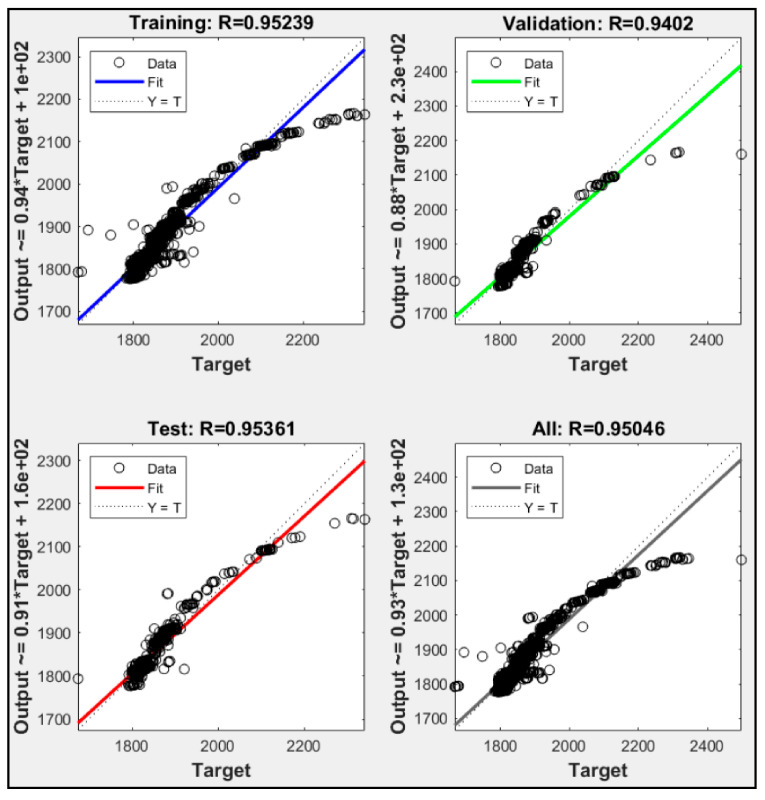
Regression result of P3.

Utilizing only one parameter, each model failed to execute until ten thousand iterations. Each model always met the maximum validation check. P2 had the highest iteration check among them with 1534 iteration, while P1 and P3 were only able to execute 1031 and 1025 iteration, respectively. Figure 5 shows training data for P1, P2 and P3, while Figure 6, Figure 7 and Figure 8 shows their regression. P1 and P3 show R^2^ for all data of 0.925 and 0.954, respectively. P2 only resulted in R^2^ 0.048. With low R^2^ results from these ANN models, we could see that with only one parameter, the ANN did not meet the expectation of predicting the heat rate. Moreover, with only CO_2_ percentage as an input parameter, it could not predict the power plant heat rate.

### 3.2. Two Input Parameters

**Figure 9:**
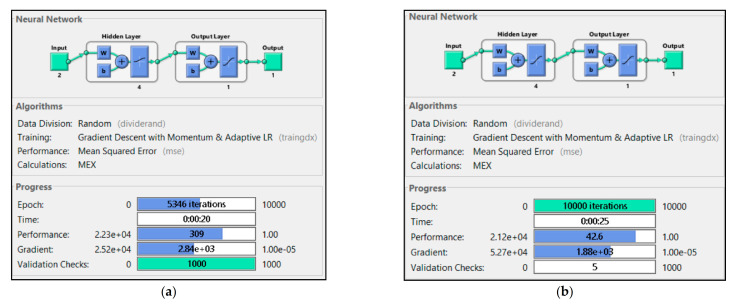

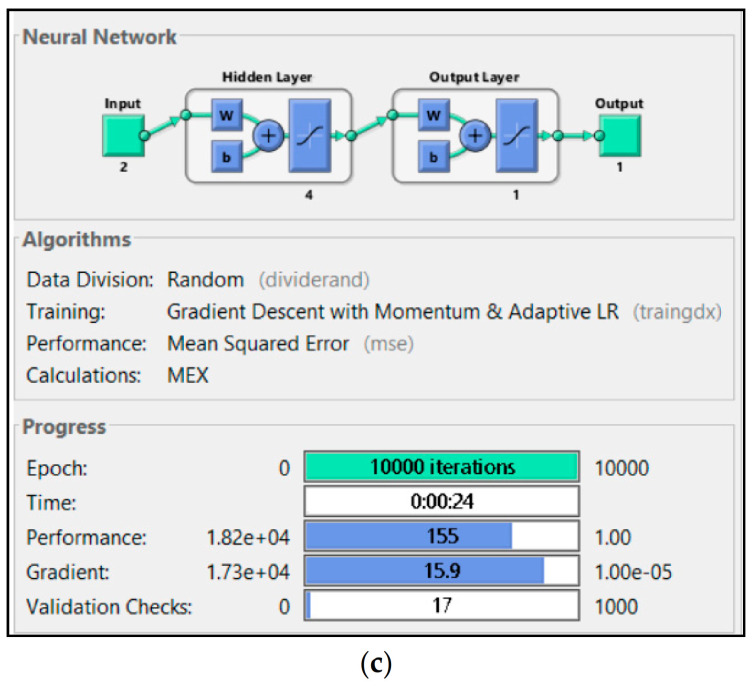
Training results of (**a**) P1 + P2, (**b**) P1 + P3 and (**c**) P2 + P3.

**Figure 10:**
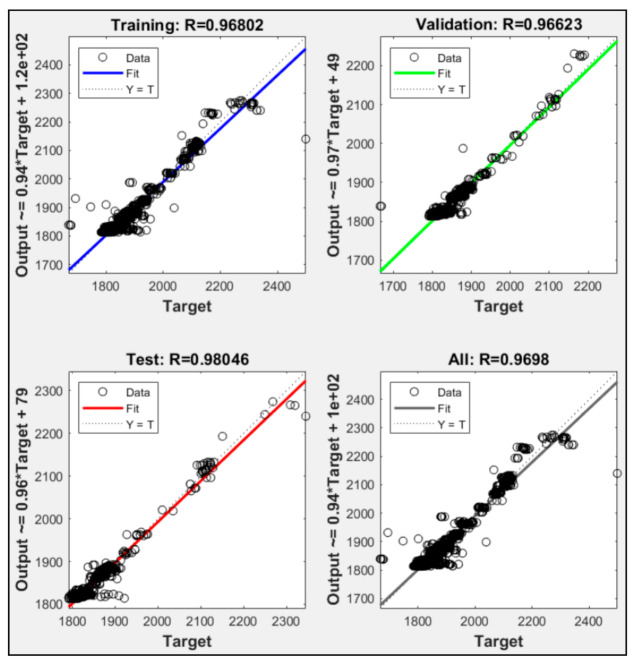
Regression result of P1 + P2.

**Figure 11:**
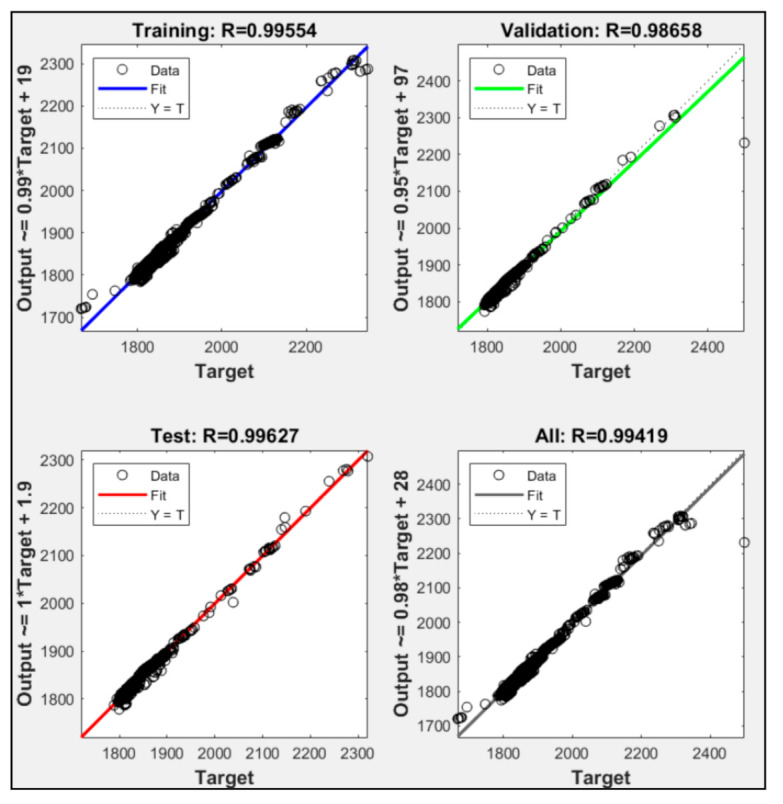
Regression result of P1 + P3.

**Figure 12:**
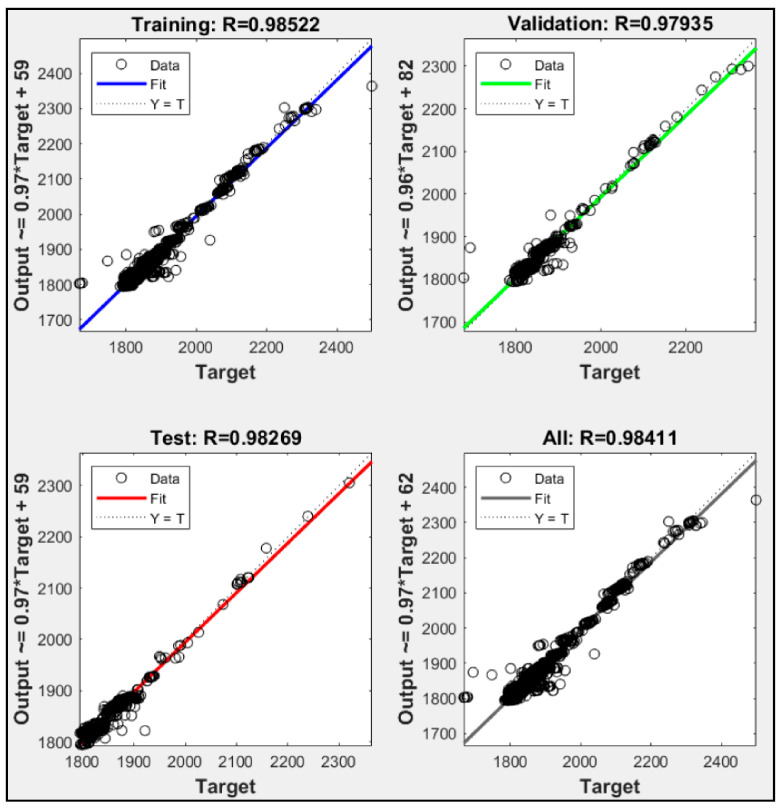
Regression result of P2 + P3.

According to the training results in Figure 9, two input parameters were used in model P1 + P2, P1 + P3, and P2 + P3. P1 + P2 failed to execute 10,000 iteration to reach the maximum validation check allowance, while P1 + P3 and P2 + P3 successfully executed up to 10,000 iterations. P1 + P2 was only able executed up to 5346 iterations. Figure 10, Figure 11 and Figure 12 show the model P1 + P2, P1 + P3, and P2 + P3 regressions R^2^ were 0.970, 0.994, and 0.984, respectively. It was shown that utilized fuel gas input and power output could predict the power plant heat rate more accurately than fuel gas input with CO_2_ percentage or CO_2_ percentage with power output. 

### 3.3. Three Input Parameters

**Figure 13:**
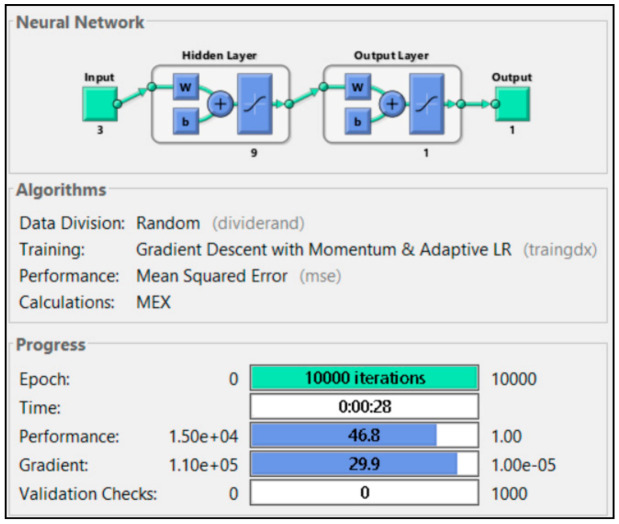
Training result of P1 + P2 + P3.

**Figure 14:**
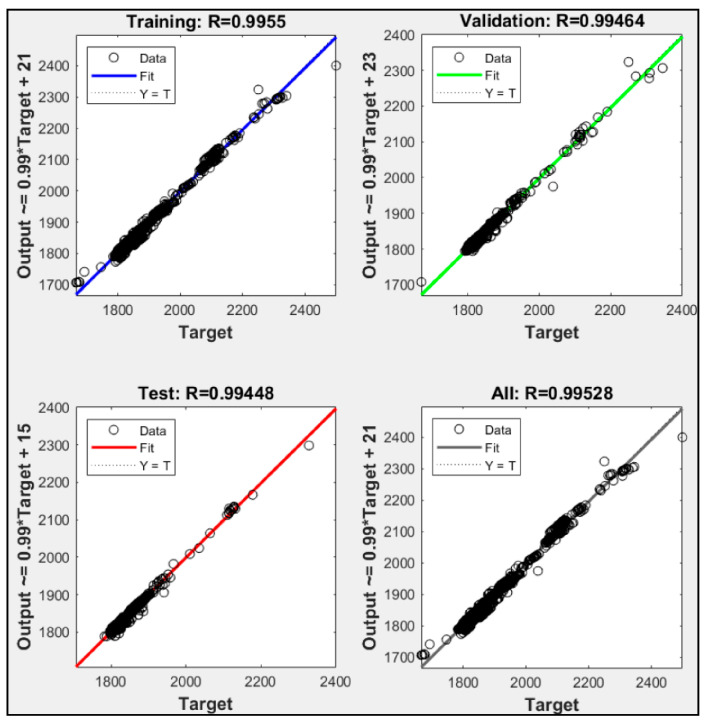
Regression of P1 + P2 + P3.

All three input parameters are combined in this section, and the training results are presented in Figure 13. Ten thousand iterations were successfully executed with zero validation checks. Figure 14 shows that the regression R^2^ for model g was 0.995, which was the highest number among all models. Based on these results, utilizing fuel gas input, CO_2_ percentage and power output together as input parameters in ANN could lead to more accurate power plant heat rate prediction.

**Figure 15:**
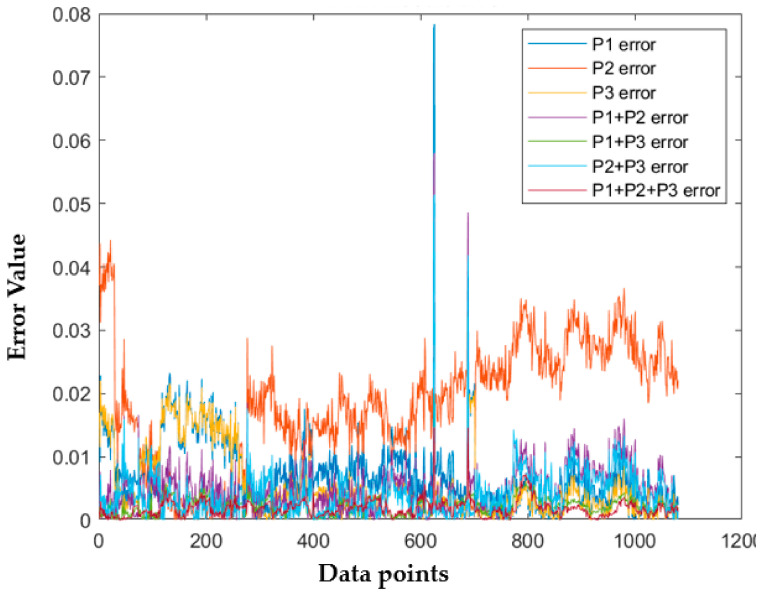
ANN result error.

Twenty five percent of 4322 data were used as test data to evaluate the ANN model performance. As many as 1081 data, as shown in Table 3 and Table 4, were obtained, and the absolute error percentage was used to see which data had the lowest error numbers. As shown in Table 5, P1 + P2 + P3 had the lowest average error with 0.19%, and P2 had the highest average error with 1.84%. P1 maximum error was the highest as shown in Figure 15 and Table 4 with 7.83%, and the maximum error of P1 + P2 + P3 was the lowest with 1.4681%.

## 4. Discussion

The experimental results showed that ANN could predict CCPP heat rate performance with correct input parameters. Those models with one input parameter were not able to execute 10,000 epoch iterations. The ANN always met the maximum validation check allowances. Combining two parameters could lead to a better heat rate prediction. P1 + P3 that used fuel gas heat input and power output was a more accurate model than the other models with two input parameters. P1 + P2 was the only two parameter input that did not meet the iteration expected. The training stopped at 5346 iterations because the validation check met the maximum allowance. The combination of P2 + P3 could meet the expected iteration. However, the R^2^ regression was only 0.984, and the validation check was 17. Combining three input parameters, P1 + P2 + P3, could produce better accuracy. Table 6 shows that P1 + P2 + P3 led to the highest regression R^2^ value. It also had a zero validation check. A validation check refers to the number of errors from the dataset. Lower validation checks could lead to higher accuracy from the model to predict the desired data. The experiment data showed that adding CO_2_ percentage as an input parameter could lead to higher accuracy in predicting CCPP heat rate value. All the variation had average error data lower than 2%. This indicates that the NN could predict heat rate accurately, and the P1 + P2 + P3 with the lowest error data could be the best variation to predict heat rate.

Input parameter, P1 + P2 + P3 as listed in Table 5, were used in ANN method to predict the actual heat rate as presented in Table 6 with 1028 data predictions. The average error for prediction was 2.52% with max error value 10.88%

## 5. Conclusions

Based on our experiment, the conclusions obtained are as follows:

- Utilizing one parameter as an input could not bring an accurate heat rate prediction. The ANN regression R^2^ value for fuel gas energy (MMBTU), CO_2_ percentage (%), and power output (MW) were 0.925, 0.005, and 0.954, respectively.
- The regression R^2^ values for two input parameters, where fuel gas energy (MMBTU) + CO_2_ percentage (%), fuel gas energy (MMBTU) + Power (MW), and CO_2_ percentage (%) + Power (MW), were 0.970, 0.994, and 0.984, respectively.
- The combination of all three parameters showed the best result of the ANN model. The regression R^2^ value was 0.995 with zero validation check and the lowest average error data.
- The three parameter combination resulted in the lowest average error in experimental data with 0.19%
- Utilizing fuel gas energy (MMBTU), CO_2_ percentage (%), and power output (MW) as inputs can lead to an accurate CCPP heat rate prediction.

This experiment was successful given that an accurate CCPP heat rate prediction was made with three input parameters. Adding more parameters like GT model, equivalent operating hours, trip history, or other parameters, might lead to a more accurate and more widely applicable ANN in predicting CCPP heat rate.

## Figures and Tables

**Table 1:** Actual operation data used.

No	Fuel Gas Heat Input (MMBTU) ^1^	CO_2_ Percentage (%)	Power Output (MW)	Heat Rate (kcal/kWh)
1	2197.821	3.678	302.52	1878.26
2	2197.821	3.678	302.52	1878.26
3	2197.821	3.678	302.88	1876.03
4	2197.821	3.678	303.21	1873.99
5	2196.798	3.636	303.09	1874.17
6	2196.798	3.636	302.34	1878.82
7	2197.423	3.563	302.73	1876.23
8	2197.423	3.563	303	1874.56
9	2200.318	3.614	303.03	1876.92
10	2202.682	3.577	303.12	1876.68
…	…	…	…	…
4313	2165.601	3.719	305.01	1816.48
4314	2165.601	3.7190	303.66	1824.56
4315	2165.601	3.7190	304.17	1821.5
4316	2165.601	3.7190	304.68	1818.45
4317	2157.497	3.595	303.87	1816.67
4318	2155.63	3.716	302.85	1820.62
4319	2155.63	3.716	303.3	1817.92
4320	2151.122	3.901	302.07	1823.05
4321	2150.759	3.804	301.86	1823.44
4322	2150.759	3.804	302.28	1820.91

^1^ Milion British Thermal Unit.

**Table 2:** Input and output parameter for the artificial neural network (ANN) model.

Group	Variation	Parameter Description	Output Parameter
One input Parameter	P1	Fuel gas energy (MMBTU)	heat rate
	P2	CO_2_ percentage (%)	heat rate
	P3	Power (MW)	heat rate
Two input Parameters	P1 + P2	Fuel gas energy (MMBTU) & CO_2_ percentage (%)	heat rate
	P1 + P3	Fuel gas energy (MMBTU) & Power (MW)	heat rate
	P2 + P3	CO_2_ percentage (%) & Power (MW)	heat rate
Three input parameters	P1 + P2 + P3	Power (MW) & Fuel gas energy (MMBTU)	heat rate

**Table 3:** Test data for P1, P2, P3 and P1 + P2.

No	Actual	P1	Error	P2	Error	P3	Error	P1 + P2	Error
1	1930.12	1961.38	1.62%	1858.55	3.71%	1962.81	1.69%	1928.67	0.08%
2	1943.56	1961.38	0.92%	1858.55	4.37%	1965.96	1.15%	1928.67	0.77%
3	1918.19	1962.03	2.29%	1858.32	3.12%	1960.44	2.20%	1929.10	0.57%
4	1930.22	1962.03	1.65%	1858.32	3.72%	1963.29	1.71%	1929.10	0.06%
5	1927.70	1962.03	1.78%	1858.32	3.60%	1962.69	1.82%	1929.10	0.07%
6	1929.72	1962.03	1.67%	1858.32	3.70%	1963.17	1.73%	1929.10	0.03%
7	1933.59	1962.41	1.49%	1858.15	3.90%	1964.12	1.58%	1929.30	0.22%
8	1928.04	1962.41	1.78%	1858.15	3.63%	1962.81	1.80%	1929.30	0.07%
9	1936.64	1962.41	1.33%	1858.15	4.05%	1964.83	1.46%	1929.30	0.38%
10	1927.54	1962.41	1.81%	1858.15	3.60%	1962.69	1.82%	1929.30	0.09%
1072	1816.48	1811.50	0.27%	1860.87	2.44%	1814.07	0.13%	1821.54	0.28%
1073	1824.56	1811.50	0.72%	1860.87	1.99%	1816.74	0.43%	1821.54	0.17%
1074	1821.50	1811.50	0.55%	1860.87	2.16%	1815.73	0.32%	1821.54	0.00%
1075	1818.45	1811.50	0.38%	1860.87	2.33%	1814.72	0.21%	1821.54	0.17%
1076	1816.67	1814.29	0.13%	1860.40	2.41%	1816.32	0.02%	1822.70	0.33%
1077	1820.62	1814.93	0.31%	1860.86	2.21%	1818.34	0.13%	1822.98	0.13%
1078	1817.92	1814.93	0.16%	1860.86	2.36%	1817.45	0.03%	1822.98	0.28%
1079	1823.05	1816.47	0.36%	1861.58	2.11%	1819.88	0.17%	1823.71	0.04%
1080	1823.44	1816.60	0.38%	1861.20	2.07%	1820.30	0.17%	1823.74	0.02%
1081	1820.91	1816.60	0.24%	1861.20	2.21%	1819.47	0.08%	1823.74	0.16%

**Table 4:** Test data for P1 + P3, P2 + P3 and P1 + P2 + P3.

No	Actual		P1 + P3	Error	P2 + P3	Error	P1 + P2 + P3	Error
1	1930.12		1930.85	0.04%	1935.11	0.26%	1932.78	0.14%
2	1943.56		1938.25	0.27%	1939.50	0.21%	1943.22	0.02%
3	1918.19		1924.42	0.32%	1932.04	0.72%	1923.87	0.30%
4	1930.22		1931.08	0.04%	1935.93	0.30%	1933.33	0.16%
5	1927.70		1929.69	0.10%	1935.12	0.38%	1931.35	0.19%
6	1929.72		1930.80	0.06%	1935.77	0.31%	1932.94	0.17%
7	1933.59		1932.52	0.06%	1937.24	0.19%	1935.61	0.10%
8	1928.04		1929.45	0.07%	1935.46	0.38%	1931.22	0.16%
9	1936.64		1934.19	0.13%	1938.21	0.08%	1938.01	0.07%
10	1927.54		1929.17	0.08%	1935.29	0.40%	1930.82	0.17%
1072		1816.48	1821.28	0.26%	1820.62	0.23%	1820.14	0.20%
1073		1824.56	1827.36	0.15%	1822.06	0.14%	1826.51	0.11%
1074		1821.50	1825.09	0.20%	1821.52	0.00%	1824.12	0.14%
1075		1818.45	1822.79	0.24%	1820.98	0.14%	1821.71	0.18%
1076		1816.67	1821.52	0.27%	1822.22	0.31%	1820.36	0.20%
1077		1820.62	1825.02	0.24%	1822.91	0.13%	1824.36	0.21%
1078		1817.92	1822.98	0.28%	1822.44	0.25%	1822.22	0.24%
1079		1823.05	1825.82	0.15%	1823.51	0.02%	1825.79	0.15%
1080		1823.44	1826.54	0.17%	1823.79	0.02%	1826.29	0.16%
1081		1820.91	1824.65	0.21%	1823.35	0.13%	1824.34	0.19%

**Table 5:** Test result error summary.

Input Parameter	Max Error	Average Error
P1	7.84%	0.73%
P2	4.81%	1.84%
P3	5.42%	0.58%
P1 + P2	5.80%	0.49%
P1 + P3	2.10%	0.23%
P2 + P3	5.14%	0.41%
P1 + P2 + P3	1.47%	0.19%

**Table 6:** CCPP heat rate actual prediction with ANN.

No	Actual	Prediction	Error
1	2462.34	2404.59	2.35%
2	2429.13	2436.05	0.28%
3	2461.90	2433.80	1.14%
4	2446.28	2431.91	0.59%
5	2443.30	2414.70	1.17%
6	2433.57	2431.62	0.08%
7	2460.70	2434.82	1.05%
8	2462.24	2438.58	0.96%
9	2436.63	2451.69	0.62%
10	2447.93	2437.34	0.43%
1019	2447.89	2447.84	0.00
1020	2451.29	2430.37	0.01
1021	2445.20	2444.25	0.00
1022	2439.77	2394.99	0.02
1023	2420.73	2431.86	0.00
1024	2453.71	2437.44	0.01
1025	2423.18	2422.99	0.00
1026	2409.58	2432.12	0.01
1027	2439.65	2412.53	0.01
1028	2431.93	2421.86	0.00

## References

[1] Wood D.A. (2020). Combined cycle gas turbine power output prediction and data mining with optimized data matching algorithm. SN Appl. Sci..

[2] Liu Z., Karimi I.A. (2020). Gas turbine performance prediction via machine learning. Energy.

[3] Bartolini C., Caresana F., Comodi G., Pelagalli L., Renzi M., Vagni S. (2011). Application of artificial neural networks to micro gas turbines. Energy Convers. Manag..

[4] Anvari S., Taghavifar H., Saray R.K., Khalilarya S., Jafarmadar S. (2015). Implementation of ANN on CCHP system to predict trigeneration performance with consideration of various operative factors. Energy Convers. Manag..

[5] Fast M., Assadi M., De S. (2009). Development and multi-utility of an ANN model for an industrial gas turbine. Appl. Energy.

[6] Rossi F., Velázquez D., Monedero I., Biscarri F. (2014). Artificial neural networks and physical modeling for determination of baseline consumption of CHP plants. Expert Syst. Appl..

[7] Elfaki E.A., Hassan A.H. (2018). Prediction of electrical output power of combined cycle power plant using regression ANN model. J. Power Energy Eng..

[8] Tüfekci P. (2014). Prediction of full load electrical power output of a base load operated combined cycle power plant using machine learning methods. Int. J. Electr. Power Energy Syst..

[9] Rua Orozco D.J., Venturini O.J., Escobar Palacio J.C., del Olmo O.A. (2017). A new methodology of thermodynamic diagnosis, using the thermoeconomic method together with an artificial neural network (ANN): A case study of an externally fired gas turbine (EFGT). Energy.

[10] Plis M., Rusinowski H. (2018). A mathematical model of an existing gas-steam combined heat and power plant for thermal diagnostic systems. Energy.

[11] Palme T., Breuhaus P., Assadi M., Klein A., Kim M. (2011). New Alstom Monitoring Tools Leveraging Artificial Neural Network Technologies. ASME Turbo Expo.

[12] Smrekar J., Pandit D., Fast M., Assadi M., De S. (2009). Prediction of power output of a coal-fired power plant by artificial neural network. Neural Comput. Appl..

[13] Fantozzi F., Desideri U. (1998). Simulation of power plant transients with artificial neural networks: Application to an existing combined cycle. Proc. Inst. Mech. Eng..

[14] Flynn D., Ritchie J., Cregan M. (2005). Data Mining Techniques Applied to Power Plant Performance Monitoring. IFAC Proceedings Volumes.

[15] Lu S., Hogg B. (2000). Dynamic nonlinear modelling of power plant by physical principles and neural networks. Int. J. Electr. Power Energy Syst..

[16] Pan L., Flynn D., Cregan M. Statistical Model for Power Plant Performance Monitoring and Analysis. Proceedings of the 2007 42nd International Universities Power Engineering Conference.

[17] Khudair O.A., Abass K.A., Abed N.S., Ali K.H., Abdulaziz S., Shaboot A.C. (2018). Theoretical Investigation For The Effect of Fuel Quality on Gas Turbine Power Plants. J. Phys. Conf. Ser..

[18] Walzcak S., Narciso C. Artificial Neural Network. https://www.researchgate.net/publication/288156361_Artificial_Neural_Networks.

[19] Taghavifar H., Mardani A., Taghavifar L. (2013). A hybridized artificial neural network and imperialist competitive algorithm opti-mization approach for prediction of soil compaction in soil bin facility. Measurement.

[20] ASME ASME PTC 22—2005 Gas Turbine Performance Test Code. https://www.twirpx.com/file/866220/.

